# SDF-1α/CXCR4 Axis Mediates The Migration of Mesenchymal Stem Cells to The Hypoxic-Ischemic Brain Lesion in A Rat Model

**DOI:** 10.22074/cellj.2015.504

**Published:** 2015-01-13

**Authors:** Qin Yu, Lizhen Liu, Jie Lin, Yan Wang, Xiaobo Xuan, Ying Guo, Shaojun Hu

**Affiliations:** 1 College of Life Science, Zhejiang Chinese Medical University, Hangzhou, China; 2 Institute of Bioengineering, Zhejiang Chinese Medical University, Hangzhou, 310053, China; 3 Bone Marrow Transplantation Center, The First Affiliated Hospital, Zhejiang University School of Medicine, Hangzhou, China; 4 The First Affiliated Hospital, Zhejiang Chinese Medical University, Hangzhou, China

**Keywords:** Mesenchymal Stem Cells, Migration, SDF-1α, CXCR 4

## Abstract

**Objective:**

Transplantation of mesenchymal stem cells (MSCs) can promote functional recovery of the brain after hypoxic-ischemic brain damage (HIBD). However, the mechanism regulating MSC migration to a hypoxic-ischemic lesion is poorly understood. Interaction between stromal cell-derived factor-1α (SDF-1α) and its cognate receptor CXC chemokine receptor 4 (CXCR4) is crucial for homing and migration of multiple stem cell types. In this study, we investigate the potential role of SDF-1α/CXCR4 axis in mediating MSC migration in an HIBD model.

**Materials and Methods:**

In this experimental study, we first established the animal model of HIBD using the neonatal rat. Bone marrow MSCs were cultured and labeled with 5-bromo-21-deoxyuridine (BrdU) after which 6×10^6^ cells were intravenously injected into the rat. BrdU positive MSCs in the hippocampus were detected by immunohistochemical analyses. The expression of hypoxia-inducible factor-1α (HIF-1α) and SDF-1α in the hippocampus of hypoxic-ischemic rats was detected by Western blotting. To investigate the role of hypoxia and SDF-1α on migration of MSCs *in vitro*, MSCs isolated from normal rats were cultured in a hypoxic environment (PO_2_=1%). Migration of MSCs was detected by the transwell assay. The expression of CXCR4 was tested using Western blotting and flow cytometry.

**Results:**

BrdU-labeled MSCs were found in the rat brain, which suggested that transplanted MSCs migrated to the site of the hypoxic-ischemic brain tissue. HIF-1α and SDF-1α significantly increased in the hippocampal formations of HIBD rats in a time-dependent manner. They peaked on day 7 and were stably expressed until day 21. Migration of MSCs *in vitro* was promoted by SDF-1α under hypoxia and inhibited by the CXCR4 inhibitor AMD3100. The expression of CXCR4 on MSCs was elevated by hypoxia stimulation as well as microdosage treatment of SDF-1α.

**Conclusion:**

This observation illustrates that SDF-1α/CXCR4 axis mediate the migration of MSCs to a hypoxic-ischemic brain lesion in a rat model.

## Introduction

Hypoxic-ischemic brain damage (HIBD) remains a significant cause of neonatal mortality and longterm neurological deficits (1-3). Presently, the only available treatment, hypothermia, has limited beneficial effects and is only effective in mildly-affected children (4, 5). There is an urgent need to develop more effective therapeutic strategies.

One emerging strategy with therapeutic potential is mesenchymal stem cells (MSCs) treatment. A growing number of studies in rodent models show that MSCs transplantation significantly reduces lesion volume and improves functional outcome after HIBD (6-8). Our recent work has also demonstrated that intravenous infusion of MSCs improved learning and memory ability after HIBD. The results of previous studies showed that MSCs could survive and migrate to the brain lesions of hypoxic-ischemic rats (5-8). It therefore seems that the hypoxic-ischemic tissues can specifically attract MSCs and mediate their migratory behavior. However, the mechanisms regulating MSCs migration to the injured brain remain to be revealed.

It appears that chemokine receptors are critical for MSCs homing. Growing evidence indicates that stromal cell-derived factor-1α (SDF-1α) and its cellular receptor, CXC chemokine receptor 4 (CXCR4), play an important role in MSCs migration. SDF-1α/CXCR4 has been shown to direct the migration of stem cells associated with injury repair in many species and tissue types (9-11). Moreover, expression of both SDF-1α and CXCR4 is predominantly promoted under hypoxic conditions such as acute injury (12, 13). This raises the possibility that the SDF-1α/CXCR4 axis also plays essential roles in directing bone marrowderived MSCs migration in the hypoxic-ischemic brain tissue.

In this study, we therefore investigated the role of the SDF-1α/CXCR4 axis on migration of MSCs to the hypoxic-ischemic brain lesions in a rat model.

## Materials and Methods

### Animals

In this experimental study, neonatal male rats (7 days after birth) were purchased from Zhejiang Chinese Medical University Animal Centre. All animals were allowed free access to food and water.

All animal investigations were conducted in accordance with the Guide for the Care and Use of Laboratory Animals published by National Institutes Health (NIH) and approved by The Institutional Animal Care Committee of Zhejiang Chinese Medical University.

### Cell culture and reagents

MSCs were obtained from rat femoral and tibial bone marrow. Briefly, muscles and the entire connective tissue were detached, and the epiphyses were removed. Marrow was harvested by inserting an 18-gauge syringe needle into one end of the bone shaft and flushing the contents into a 60 mm culture dish that contained proliferation culture medium which consisted of Dulbecco’s Modified Eagle’s Medium (DMEM, Invitrogen, USA) supplemented with 10% (v/v) screened fetal bovine serum (FBS, Invitrogen, USA). A single-cell suspension was obtained by passage through needles of decreasing size. Cells were then centrifuged, nucleated cells were counted, and seeded at a density of 5×10^5^ cells/cm^2^ in culture medium at 37˚C in 5% CO_2_. After 48 hours, all non-adherent cells were removed by medium exchange. The medium was subsequently replaced every 3 days. The monolayer of adherent cells was trypsinized (0.25% trypsin-ethylene diamine tetraacetic acid, Invitrogen, USA) at 80% confluency, resuspended in culture medium, and seeded at a density of 10000 cells/cm^2^. Passages 3-6 were used in this study.

For labeling the MSCs, 5-bromo-21-deoxyuridine (BrdU, 10 μM) (Sigma-Aldrich, USA) was added to the culture medium for 48 hours.

For the hypoxia culture, we generated an atmosphere of 1% O_2_, 5% CO_2_ and 94% N_2_. MSCs were cultured in an incubator that contained the hypoxic gas.

### Animal model of HIBD and transplantation of MSCs

HIBD was induced in rats by unilateral left carotid artery ligation under anesthesia. Hypoxic brain injury (8% O_2_ for 2.5 hours) was also generated as previously described (14). Body temperature was maintained at 37˚C while the rats were inside the hypoxic chamber. BrdU-labeled MSCs were resuspended at 6×10^6^ cells/mL in normal (0.9%) saline. A 1-mL cell suspension was intravenously injected using a transfusion needle. The hippocampi of hypoxic-ischemic rats were obtained for protein detection.

### Immunohistochemical (IHC) analyses

The hippocampi of hypoxic-ischemic rats were removed and fixed in 4% paraformaldehyd prior to paraffin embedding and sectioning. Following dewaxing and rehydration, hippocampus sections were stained with hematoxylin and eosin (H&E) and immunohistochemistry performed. To detect BrdU positive cells, paraffin sections were retrieved using Target Retrieval Solution (Dako, Carpinteria, USA). After endogenous peroxidase and nonspecific protein blocking, primary antibodies were incubated overnight at 4˚C with anti-BrdU antibody (1:50, Santa Cruz, USA) and rabbit control immunoglobulin G (IgG). After washing, the immunoreactivity of the sections was analyzed using an EnVision detection kit (Dako, Denmark), according to the manufacturer’s instructions. Positive staining was brown.

### Western blot analyses

Protein from the cell lysates was mixed with 4X loading buffer that contained 10 mM dithiothreitol and boiled for 10 minutes prior to electrophoresis on 10% sodium dodecyl sulfate-polyacrylamide gels. Following transfer onto polyvinylidene fluoride membranes and blocking, the membranes were incubated overnight at 4˚C with either HIF-1α antibody (Thermo, USA), SDF-1α antibody (BioVision, USA) or CXCR4 antibody (BioVision, USA). Following several washes in tris-buffered saline and tween 20 (TBST), membranes were subsequently incubated for 1 hour with horseradish peroxidase-conjugated secondary antibody (Sigma-Aldrich, USA). The membrane was washed three times with TBST. The signals were detected by enhanced chemiluminescence reagents (Thermo, USA). The density of the bands was quantified using Image J software (National Institutes of Health).

### Migration assays

Migration assays were carried out in a sixwell transwell using polycarbonate membranes with 8 μm pores (Greiner Bio One, Frickenhausen, Germany). MSCs [2×10^5^ cells/mL in 1 mL of medium (DMEM/F-12 + 2% FBS)] were placed in the upper chamber of the transwell assembly. The lower chamber contained 1 mL of medium treated with SDF-1α or CXCR4 antagonist AMD3100. After incubation under hypoxia (1% O_2_, 5% CO_2_) for 10 hours, the upper surface of the membrane was scraped gently to remove non-migrating cells and washed with phosphate-buffered saline (PBS). The membrane was then fixed in 4% paraformaldehyde for 15 minutes and stained in 0.5% crystal violet for 10 minutes. The number of migrating cells was determined by counting five random fields per well at ×200 magnification. Experiments were carried out in triplicate.

### Flow cytometry

Cells were incubated with rabbit anti-rat CXCR4 antibody (BioVision, USA) for 30 minutes at room temperature. They were then washed and followed by 30 minutes of free-light incubation with PE conjugated goat anti-rabbit IgG (MultiScience, China) at 4℃, then washed and acquired on a flow cytometer (FACScan, Becton Dickinson, USA). The data were analyzed using the FlowJo program (Tree Star Inc., San Carlo, USA).

### Statistical analyses

Data are presented as mean ± SD. Data were analyzed by statistical package for the social sciences (SPSS) 13.0 software (Chicago, IL, USA) using analysis of variance (ANOVA). P<0.05 was considered significant.

## Results

### Migration of transplanted MSCs to brain lesions

**Fig 1:**
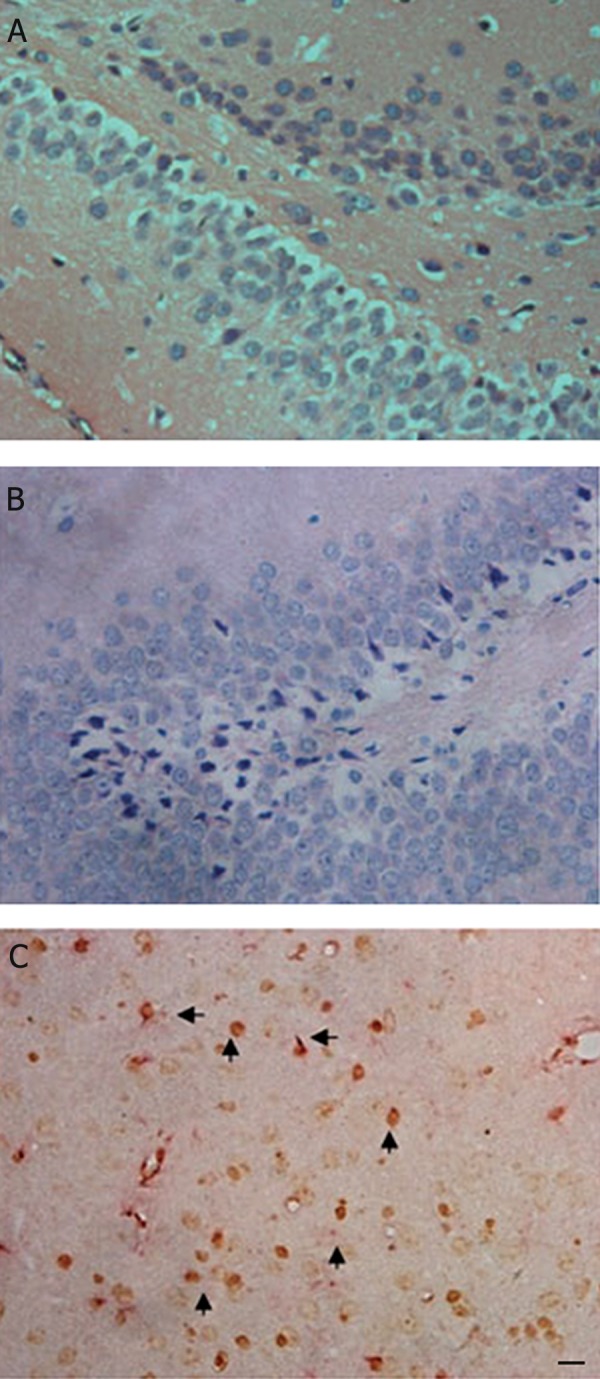
Migration of transplanted mesenchymal stem cells (MSCs) to brain lesions of the hypoxic-ischemic brain damaged (HIBD) rat. A. Hematoxylin and eosin (H&E) staining of hippocampus formation in a normal rat. B. H&E staining of hippocampus formation of the HIBD rat. Atrophic and trachychromatic neuron cells were shown in the fascia dentate of hippocampus formations and the tissue space is enlarged. C. Migration of transplanted MSCs to the hippocampi formations of HIBD rats. Immunohistochemical staining shows that the BrdU- labeled MSCs (↑) migrated into the hypoxia-ischemia brain lesions of rats (H&E ×400, scale bar=20 μm).

H&E staining showed that degeneration and necrosis of nerve cells in hippocampal formations were aggravated in rats that suffered from HIBD. After the intravenous transplantation of MSCs was accepted, BrdU-labeled MSCs were found in the rat brain (Fig 1). This observation suggested that transplanted MSCs might have the capacity for preferential migration to the site of hypoxic-ischemic brain tissue.

### Upregulated expression of HIF-1α and SDF-1α in HIBD sections

**Fig 2:**
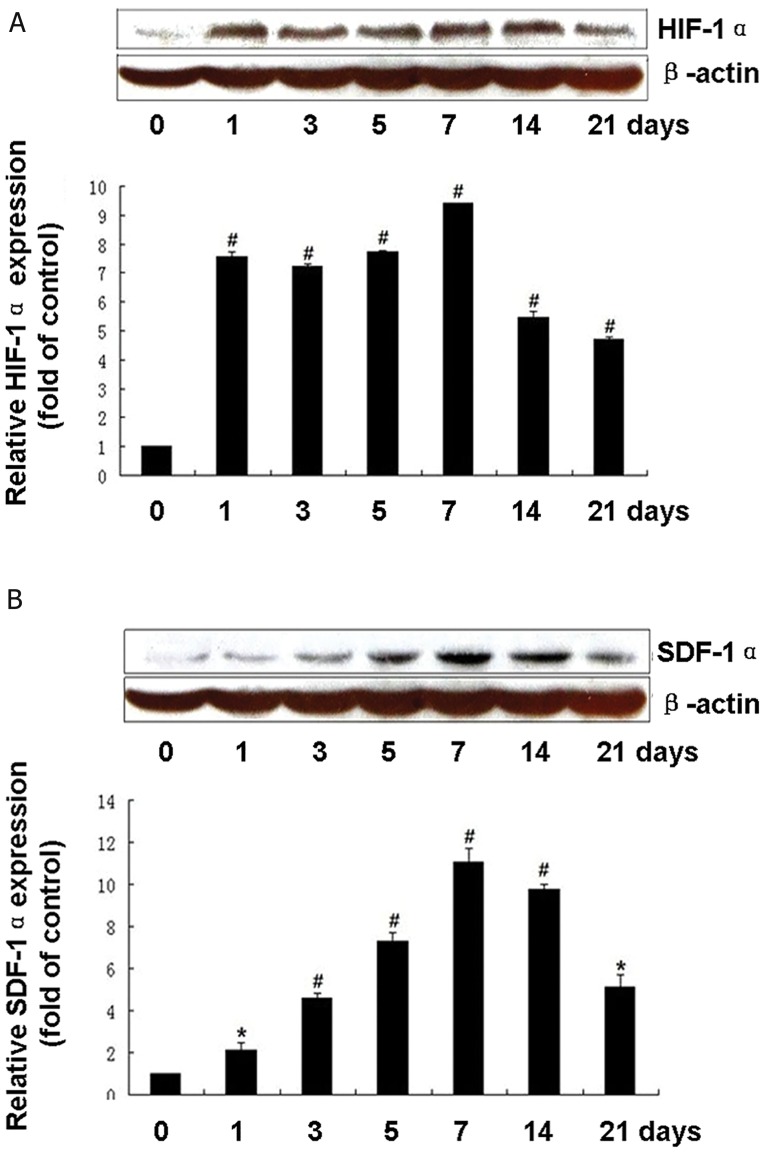
Upregulated expression of hypoxia-inducible factor-1α (HIF-1α) and stromal cell-derived factor-1α (SDF-1α) in hypoxic-ischemic brain damaged (HIBD) sections. The expressions of HIF-1α and SDF-1α in the hippocampi of HIBD rats were detected by western blotting on days 1, 3, 5, 7, 14, and 21 (n=3). *; P<0.05, #; P<0.01 vs. control. A. HIF-1α protein level in the hippo - campus of HIBD at different time points. B. Protein level of SDF-1α expressed in the hippocampi of HIBD rats.

We investigated the expression of HIF-1α and SDF-1α by western blotting in the hippocampi of rats treated by hypoxia-ischemia for 1, 3, 5, 7, 14, and 21 days. HIF-1α was stably expressed in the hippocampi and increased significantly in a timedependent manner in rats with hypoxia-ischemia in a time-dependent manner (p<0.05 vs. control), which reached a peak on day 7 (Fig 2A). As expected, the level of SDF-1α in the hippocampi of HIBD rats was higher than that of the normal control group. This level reached a peak on day 7 (p<0.01, Fig 2B). Subsequently, at 14 and 21 days after hypoxia-ischemia, the protein levels of HIF- 1α and SDF-1α were less than observed on day 7.

### In vitro study of the effect of the SDF-1α/CXCR4 axis on the migration of MSCs

**Fig 3:**
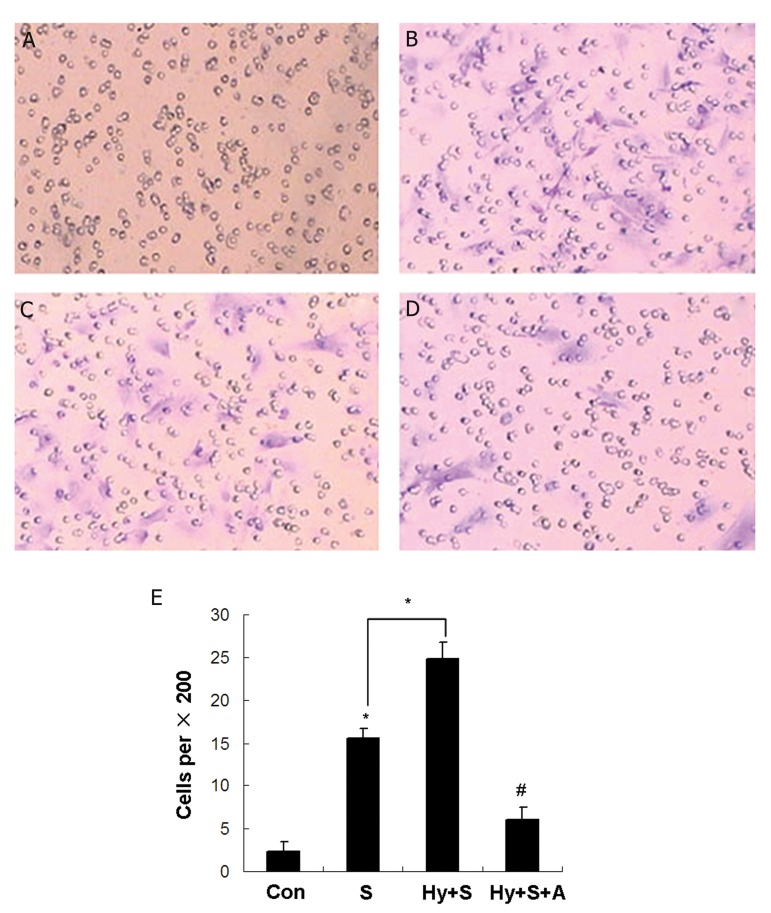
The migration of mesenchymal stem cells (MSCs) is promoted by stromal cell-derived factor-1α (SDF-1α) under hypoxia. A. Control group. B. Hypoxia+SDF-1α treatment group. C. SDF-1α treatment group. D. Hypoxia+SDF-1α+AMD3100 treatment group. E. Quantification of transwell results. Con; Control, S; SDF-1α treatment, Hy+S; SDF-1α + hypoxia treatment and Hy+S+A; Hypoxia+SDF-1α+AMD3100 treatment.

Compared to normoxic conditions (Fig 3A), MSCs migrated more rapidly in response to the chemokine SDF-1α under hypoxic conditions (Fig 3B). In addition, exposure of MSCs to SDF- 1α in normal condition increased their migration (Fig 3C). Furthermore, AMD3100 treatment decreased the migration of MSCs (Fig 3D, E). These results confirmed that SDF-1α promoted the migration of MSCs in hypoxic conditions whereas pre-treatment with a CXCR4-specific antagonist AMD3100 prevented their migration.

### Hypoxia elevates the expression of CXCR4 in MSCs

**Fig 4:**
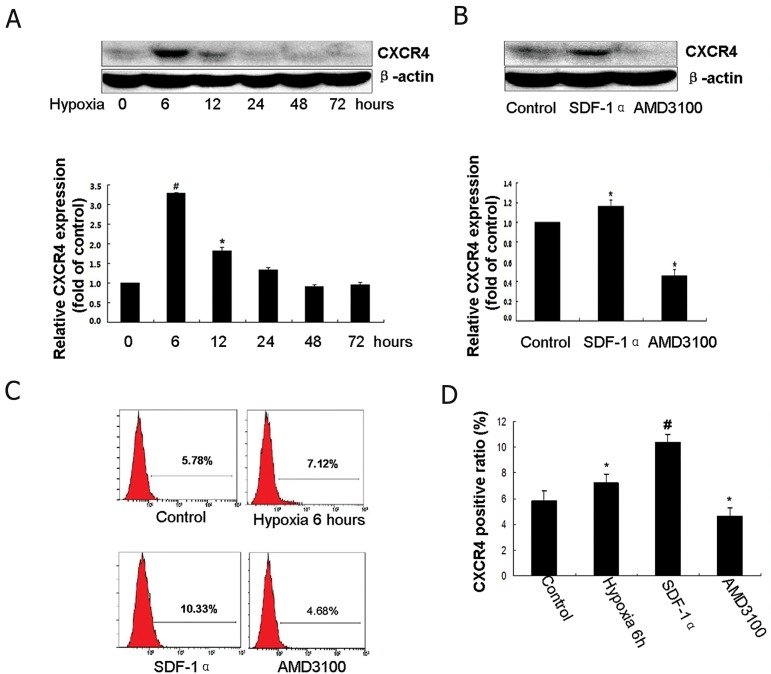
Hypoxia and a microdosage stromal cell-derived factor-1α (SDF-1α) augmented the surface expression of cognate receptor CXC chemokine receptor 4 (CXCR4) on mesenchymal stem cells (MSCs). A. After treatment with hypoxia for 0, 6, 12, 24, 48 and 72 hours, expression of CXCR4 was detected by western blotting. *; P<0.05, #; P<0.01 vs. control. B. MSCs were incubated with SDF-1α (10 ng/mL) or AMD3100 (5 μg/mL); expression of CXCR4 was detected by western blotting. C. The results of CXCR4 expression detected by flow cytometry. D. Quantification of flow cytometry results.

After treatment with hypoxia for 0, 6, 12, 24, 48 and 72 hours, the expression of CXCR4 on MSCs were detected by Western blotting and flow cytometry. The protein expression of CXCR4 is low or undetectable in MSCs under normal conditions. However, the CXCR4 protein level significantly upregulated in MSCs exposed to hypoxia for 6 and 12 hours (p<0.05, Fig 4A). This level peaked at 6 hours (p<0.01). Flow cytometry analysis showed a significantly higher percentage of CXCR4 positive cells in the MSCs exposed to hypoxia for 6 hours compared to the controls. These data provided evidence that expression levels of CXCR4 in MSCs were upregulated by hypoxia.

### A microdosage SDF-1α augments the surface expression of CXCR4 on MSCs

We used Western blotting and flow cytometry to detect the effect of SDF-1α on the surface expression of CXCR4 on MSCs. As expected, CXCR4 expression was highly upregulated in the SDF-1α (10 ng/ ml)-treated group compared with the normal control group (p<0.01). In the AMD3100-treated group CXCR4 decreased markedly. These results were confirmed by measurement of the protein level and by flow cytometry (p<0.05, Fig 4B-D).

## Discussion

MSCs have been assumed to exhibit homing features that facilitate their ability to migrate and engraft into an injured tissue and mediate repair (15). This ability of implanted MSCs to migrate to the site of damaged tissue has been confirmed in bone or cartilage fractures, myocardial infarction and ischemic cerebral injury (9, 16, 17). In this study, we also observed that transplanted MSCs could migrate and home to hypoxic-ischemic brain tissue.

Among the many environmental signals in ischemic tissue that may be involved in the recruitment of progenitor cells, the farthest upstream is hypoxia. The transcription factor hypoxia-inducible factor-1 (HIF-1) is a central regulator that expresses in response to hypoxia which mediates systemic homeostatic responses to low levels of oxygen (18). HIF-1 is a heterodimeric protein composed of a constitutively expressed HIF-1β subunit and an O_2_-regulated HIF-1α subunit. Under normoxic conditions the HIF-1α subunit is ubiquitinated and degraded. Under hypoxic conditions HIF-1α accumulates, dimerizes with HIF- 1β, and activates the transcription of downstream target genes encoding multiple angiogenic growth factors and cytokines of potential importance in wound healing (19). In this study, we have observed that HIF-1α increased in the hippocampus of the rat model following HI brain damage.

MSCs migration may involve various chemokines, cyokines, and integrins. Among the chemokines and their corresponding receptors, the SDF-1α/ CXCR4 axis is the most extensively studied system (20-22). SDF-1α, a member of the CXC subfamily, is widely expressed in many organs. The ischemia microenvironment in the injured tissues can up-regulate its expression. We have observed increased SDF-1α expression in the current study. There is report indicated that SDF-1α gene expression is regulated by HIF-1, resulting in selective in vivo expression of SDF-1α in ischemic tissue in direct proportion to reduced oxygen tension. In addition, HIF-1-induced SDF-1α expression increases the adhesion, migration and homing of circulating CXCR4-positive progenitor cells to ischemic tissue (23). We propose that HIF-1-induced SDF-1α expression also increases the homing of injected MSCs to the injured brain tissue. Interestingly, both SDF-1α and hypoxia are present in the bone marrow niche (24, 25), suggesting that hypoxia may be a fundamental requirement for progenitor cell trafficking and function. In the present study, we have found that SDF-1α increased significantly in the hypoxic ischemic rat in a time-dependent manner. The results suggested that SDF-1α might be an important factor in the microenvironment of the brain and trigger the migration of MSCs.

In order to further determine the mechanism of MSC migration we performed a migration assay with transwells. The number of migrated MSCs in the SDF-1α-treated group was considerably more than that of the negative control group and AMD3100-antagonist group. Our results confirmed that SDF-1α promoted the migration of MSCs, particularly under hypoxic conditions. Although similar results have been reported (9-12), these results consolidated the role of the SDF-1α/ CXCR4 axis on the migration of MSCs to the impaired site in the brain.

CXCR4, one of the specific receptors of SDF- 1α, is expressed both on the cell surface and inside MSCs, where it normally exists. A number of studies have demonstrated that CXCR4 expression on MSCs can be promoted by hypoxia (26-28). In this study, our results indicated that both hypoxia and SDF-1α stimulated the expression of CXCR4. The increased SDF-1α in the injured tissue could cause it to mobilize to the cell surface. This translocated surface CXCR4 binds to SDF-1α and directs the migration of MSCs toward the injured tissue.

## Conclusion

The present study suggests that the SDF-1α/ CXCR4 axis mediates the migration of MSCs to the hypoxic-ischemic brain lesion in a rat model. These results provide a novel insight into the mechanisms responsible for MSC migration, and may be of help to improve the transplantation efficiency of MSCs in the future.
